# Frequency of pregnant HBV/HCV carriers and status of their internal medicine consultation: A nationwide survey in Japan

**DOI:** 10.1111/jog.16351

**Published:** 2025-06-29

**Authors:** Manabu Ogoyama, Shunji Suzuki, Shin‐ichi Hoshi, Akihiko Sekizawa, Yoko Sagara, Isamu Ishiwata, Tadaichi Kitamura

**Affiliations:** ^1^ Division of Maternal and Child Health Japan Association of Obstetricians and Gynecologists Tokyo Japan; ^2^ Infectious Diseases Japanese Foundation for Sexual Health Medicine Tokyo Japan

**Keywords:** hepatitis B virus, hepatitis C virus, hepatitis virus carrier, internal medicine consultation, pregnancy

## Abstract

**Aim:**

The present study investigated the prevalence of pregnant hepatitis B (HBV) and hepatitis C (HCV) carriers and their internal medicine consultation rates in Japan.

**Methods:**

A questionnaire was sent to 1931 delivery facilities regarding their delivery management policies and care of pregnant HBV and HCV carriers who delivered between January and December 2023, including internal medicine consultation rates.

**Results:**

Responses were received from 1077 facilities (55.8%). The prevalence of HBV was 0.14% in Japanese women (406/288847) and 3.00% (196/6527) in non‐Japanese women. The prevalence of HBV in 12 Japanese teenagers (≤19 years) was 0.53%, which was significantly higher than that in adult pregnant women (0.14%) (*p* < 0.001). HBe antigen was positive in 12.6% of pregnant HBV carriers. The overall prevalence of HCV was 0.23% (684/295374), with similar rates in Japanese and non‐Japanese women (0.23% vs. 0.31%), and 3.1% (21/684) had high HCV‐RNA levels. Internal medicine consultations were performed on 64.8% (295/455) of known HBV carriers before pregnancy and on 66.7% (98/147) of those diagnosed during pregnancy. Regarding HCV, consultation rates were 10.5% (68/645) before pregnancy and 69.2% (27/39) during pregnancy.

**Conclusions:**

The prevalence of pregnant hepatitis virus carriers in Japan is approximately 0.2% for both HBV and HCV, with a high HBV rate in teenagers. Only 60%–70% of patients receive proper internal medicine consultations, highlighting the need for increased awareness among patients, physicians, and healthcare systems.

## INTRODUCTION

In recent years, the introduction of direct‐acting antivirals (DAAs) for hepatitis B virus (HBV) and hepatitis C virus (HCV) infections has markedly improved treatment outcomes, with a high percentage of HBV carriers achieving the suppression of viral replication and more than 95% of HCV carriers achieving viral clearance.^1^ Therefore, the early detection of hepatitis virus carriers and continuous consultation by hepatology specialists after their diagnosis are essential. Although the prevalence of pregnant HBV carriers in Japan was previously reported to be approximately 0.2%, while that of HCV carriers was around 0.1%–0.2%,^2^ the rate of internal medicine consultation for these carriers in clinical practice is suboptimal. A nationwide survey conducted by Tanaka et al. in 2016–2017 found that only approximately 78.5% of obstetricians responsible for deliveries reported that their HBV/HCV carrier patients were referred to hepatology specialists, indicating that a subset of pregnant women still do not receive appropriate follow‐ups.^3^ In response, the government has introduced a number of measures, including subsidizing the costs of the initial detailed examination (blood tests and an ultrasound examination) for pregnant women diagnosed as hepatitis virus carriers during prenatal check‐ups to increase internal medicine consultation rates and promote sustained follow‐ups for these patients. In addition, prevention of vertical transmission of hepatitis viruses is also an important issue. Current guidelines recommend the administration of antiviral therapy, tenofovir to pregnant women with high HBV‐DNA level after 28 weeks of gestation.^4^, ^5^, ^6^, ^7^ Regarding HCV, the guidelines do not generally advocate elective cesarean delivery as a preventive measure.^8^, ^9^ Therefore, the present study investigated the prevalence of pregnant HBV and HCV carriers and their internal medicine consultation rates in Japan in 2023. We then evaluated whether changes have occurred in the rates of internal medicine consultation among pregnant HBV and HCV carriers compared to those reported in previous studies conducted in 2016–2017.^3^ In addition, we investigated the current status in Japan of tenofovir administration in HBV carriers and selective cesarean delivery in HCV carriers for the prevention of vertical transmission of hepatitis viruses.

## METHODS

With the cooperation of Infectious Diseases, the Japanese Foundation for Sexual Health Medicine, the Japan Association of Obstetricians and Gynecologists (JAOG) conducted a questionnaire survey between July and November 2024. The survey targeted the representatives of 1931 delivery facilities nationwide, including 400 perinatal centers, 532 hospitals, and 999 obstetric clinics with inpatient services. The questionnaire asked the total number of deliveries (by age group) and distinction between Japanese and non‐Japanese (detailed nationality was not asked). Each facility's delivery management policy as well as the actual management of pregnant HBV and HCV carriers who delivered between January and December 2023 was asked. The questionnaire included inquiries on the number of cases (with high‐risk carriers being defined as those who were positive for HBe antigen or had high HCV‐RNA levels and management practices). In this questionnaire, HCV‐RNA ≥6.0 log IU/mL was indicated as the reference cutoff value. Facility policies were assessed based on the following: (1) whether the facility managed pregnant carriers for delivery on‐site and (2) its policy on referring pregnant carriers to internal medicine (i.e., hepatology specialists). In addition, the management status of pregnant carriers was evaluated by (1) the internal medicine consultation rate among women diagnosed as carriers before pregnancy and (2) the referral rate to internal medicine among those diagnosed as carriers for the first time during pregnancy, with analyses stratified by nationality (Japanese and non‐Japanese) and age group (≤19 years, 20–29 years, 30–39 years, and ≥40 years). In addition, the questionnaire asked the number of HBV carriers who received tenofovir, and that of HCV carriers who underwent cesarean delivery for preventing vertical infection. Statistical analyses were performed using EZR version 1.68 (Saitama Medical Center, Jichi Medical University), with comparisons between groups conducted using the chi‐square test and a *p*‐value <0.05 being significant. This survey was approved by the Ethics Committee of JAOG (Ethics Board approval number: 202407_3).

## RESULTS

Of the 1931 facilities contacted, responses were received from 1077 facilities (response rate: 55.8%). Among these, 506 facilities (47.0%) were hospitals/perinatal centers and 571 (53.0%) were obstetric clinics with inpatient services.

### Facility policies

Regarding delivery management by hospitals/perinatal centers, 99.1% (226/228) managed deliveries for pregnant HBV carriers on‐site and 97.8% (223/228) for pregnant HCV carriers. In contrast, only 52.7% (301/571) of obstetric clinics managed HBV carriers and 41.3% (236/571) managed HCV carriers on‐site (Table 1). Regarding referral policies, 97.4% (221/227) of hospitals/perinatal centers and only 64.6% (199/308) of obstetric clinics reported referring pregnant HBV carriers to internal medicine. The corresponding referral rates for HCV carriers were 96.6% (219/226) for hospitals/perinatal centers and 75.7% (193/255) for obstetric clinics (Table 1).

**TABLE 1:** Perinatal management of pregnant HBV/HCV carriers.

	HBV carriers		HCV carriers	
	Perinatal centers	Obstetrics clinics	Perinatal centers	Obstetrics clinics
Perinatal management				
Managed by their own institution, *n* (%)	226 (99.1)	301 (52.7)	223 (97.8)	236 (41.3)
Referred to another institution, *n* (%)	1 (0.4)	263 (46.1)	2 (0.9)	316 (55.3)
No response, *n* (%)	1 (0.4)	7 (1.2)	3 (1.3)	19 (3.3)
Referred to internal medicine (hepatology), *n* (%)	221 (97.4)	199 (64.6)	219 (96.9)	193 (75.7)

Abbreviations: HBV, hepatitis B virus; HCV, hepatitis C virus.

### Prevalence of pregnant carriers

The total number of deliveries at the facilities that responded to this survey was 295 374, of which 288 847 were Japanese and 6527 were non‐Japanese. The number of deliveries by age group is shown in Table 2. The overall prevalence of pregnant HBV carriers was 0.21% (602/288847) (Table 3). Among 602 pregnant HBV carriers, 455 (75.6%) had been diagnosed as carriers before pregnancy, while 147 (24.4%) were diagnosed during pregnancy (Table 4). When stratified by nationality, the prevalence of HBV was 0.14% (406/288847) among Japanese women and 3.00% (196/6527) among non‐Japanese women (Table 3). Although only 12 pregnant HBV carriers were ≤19 years, all of whom were Japanese, the prevalence of HBV in this age group was 0.53%, which was significantly higher than that in adult pregnant women (0.14%; *p* < 0.001) (Table 3). Following the exclusion of non‐Japanese women aged ≤19 years, the prevalence of HBV carriers was significantly higher in all age groups of non‐Japanese women than in Japanese women. The overall frequency of HBe antigen positivity among pregnant HBV carriers, suggestive of viral activity, was 12.6% (76/602); this rate was 11.1% (45/406) in Japanese women and 15.8% (31/196) in non‐Japanese women (*p* = 0.116) (Table 5).

**TABLE 2:** Age distribution of pregnant women giving birth, *n* (%).

Age group	Total	Japanese	Non‐Japanese
≤19	2246 (0.8)	2183 (0.8)	63 (1.0)
Hospital	1609	1553	56
Clinic	637	630	7
20–29	92 107 (31.2)	89 500 (31.0)	2607 (39.9)
Hospital	57 614	55 509	2105
Clinic	34 493	33 991	502
30–39	176 997 (59.9)	173 488 (60.1)	3509 (53.8)
Hospital	120 757	117 865	2892
Clinic	56 240	55 623	617
≥40	24 024 (8.1)	23 676 (8.2)	348 (5.3)
Hospital	18 994	18 694	300
Clinic	5030	4982	48
Total	295 374	288 847	6527
Hospital	198 874	193 621	5353
Clinic	96 400	95 226	1174

**TABLE 3:** Frequency of pregnant HBV and HCV carriers, *n* (%).

Age group	HBV carrier			HCV carrier		
	Total	Japanese	Non‐Japanese	Total	Japanese	Non‐Japanese
≤19	12 (0.53)	12 (0.55)	0 (0)	5 (0.22)	3 (0.14)	2 (3.17)
20–29	161 (0.17)	94 (0.11)	67 (2.57)	221 (0.24)	213 (0.24)	8 (0.31)
30–39	342 (0.19)	225 (0.13)	117 (3.33)	408 (0.23)	400 (0.23)	8 (0.23)
≥40	87 (0.36)	75 (0.32)	12 (3.45)	50 (0.21)	48 (0.20)	2 (0.57)
Total	602 (0.21)	406 (0.14)	196 (3.00)	684 (0.23)	664 (0.23)	20 (0.31)

Abbreviations: HBV, hepatitis B virus; HCV, hepatitis C virus.

**TABLE 4:** Rate of carriers who were diagnosed for the first time during pregnancy among all pregnant HBV and HCV carriers, *n* (%).^a^

Age group	HBV carrier			HCV carrier		
	Total	Japanese	Non‐Japanese	Total	Japanese	Non‐Japanese
≤19	1 (8.3)	1 (8.3)	NA	1 (20.0)	1 (33.3)	0 (0)
20–29	61 (37.9)	27 (28.7)	34 (50.7)	15 (6.8)	11 (5.2)	4 (50.0)
30–39	70 (20.4)	38 (16.9)	32 (27.4)	20 (4.9)	18 (4.5)	2 (25.0)
≥40	15 (17.2)	14 (18.7)	1 (8.3)	3 (6.0)	3 (6.3)	0 (0)
Total	147 (24.4)	80 (19.7)	67 (34.2)	39 (5.7)	33 (5.0)	6 (30.0)

Abbreviations: HBV, hepatitis B virus; HCV, hepatitis C virus; NA, not available (no carriers).

^a^ The percentage indicates the number of carriers diagnosed as carriers for the first time during pregnancy, divided by the total number of pregnant carriers in HBV and HCV, respectively.

**TABLE 5:** Frequency of high‐risk carriers, *n* (%).

Age group	HBe antigen‐positive pregnant HBV carriers			High HCV‐RNA levels in pregnant HCV carriers^a^		
	Total	Japanese	Non‐Japanese	Total	Japanese	Non‐Japanese
≤19	0 (0)	0 (0)	NA	2 (40.0)	2 (66.7)	0 (0)
20–29	30 (18.6)	12 (12.8)	18 (26.9)	8 (3.6)	7 (3.3)	1 (12.5)
30–39	44 (12.9)	31 (13.8)	13 (11.1)	8 (2.0)	8 (2.0)	0 (0)
≥40	2 (2.3)	2 (2.7)	0 (0)	3 (6.0)	3 (6.3)	0 (0)
Total	76 (12.6)	45 (11.1)	31 (15.8)	21 (3.1)	20 (3.0)	1 (5.0)

Abbreviations: HBV, hepatitis B virus; HCV, hepatitis C virus; NA, not available (no carriers).

^a^ In this survey, HCV‐RNA ≥6.0log IU/mL is reference cut‐off value.

The overall prevalence of pregnant HCV carriers was 0.23% (684/295374); it was 0.23% (664/288847) in Japanese women and 0.31% (20/6527) in non‐Japanese women (Table 3). In contrast to HBV, no significant differences were observed in the prevalence of HCV carriers between Japanese and non‐Japanese women or among the age groups examined. Among pregnant HCV carriers, 5.7% (39/684) were diagnosed for the first time during pregnancy; 5.0% (33/664) were Japanese and 30% (6/20) were non‐Japanese (Table 4). The overall percentage of pregnant HCV carriers with high HCV‐RNA levels (reference values used in this survey: ≥6.0log IU/mL) was 3.1% (21/684); it was 3.0% (20/669) in Japanese women and 5.0% (1/20) in non‐Japanese women (Table 5). In the Japanese group, three pregnant women aged ≤19 years were HCV carriers, two of whom had high HCV‐RNA levels.

### Internal medicine consultation rates

Among women known to be HBV carriers before pregnancy, 64.8% (295/455) had received an internal medicine consultation (Table 6). Among teenage‐pregnant HBV carriers diagnosed before pregnancy, only 1 of 12 (9.1%) received an internal medicine, while the consultation rate in the 20–29‐year age group was 53.0% (53/100), which was lower than those in the older age groups. Among women diagnosed as HBV carriers during pregnancy, 66.7% (98/147) were subsequently referred to internal medicine. The overall internal medicine consultation rate among women known to be HCV carriers before pregnancy was 10.5% (68/645) (Table 6). Among women diagnosed as HCV carriers during pregnancy, 69.2% (27/39) were referred to internal medicine.

**TABLE 6:** Status of medical consultations for pregnant HBV/HCV carriers, *n* (%).

Age group	HBV carriers			HCV carriers		
	Total (*n* = 602)	Diagnosed before pregnancy (*n* = 455)	Diagnosed during pregnancy (*n* = 147)	Total (*n* = 684)	Diagnosed before pregnancy (*n* = 645)	Diagnosed during pregnancy (*n* = 39)
≤19	2 (16.7)	1 (9.1)	1 (100)	2 (40.0)	1 (25.0)	1 (100)
20–29	90 (55.9)	53 (53.0)	37 (60.7)	23 (10.4)	12 (5.8)	11 (73.3)
30–39	245 (71.6)	190 (69.9)	55 (78.6)	55 (13.5)	42 (10.8)	13 (65.0)
≥40	56 (64.4)	51 (70.8)	5 (33.3)	15 (30.0)	13 (27.7)	2 (66.7)
Total	393 (65.3)	295 (64.8)	98 (66.7)	95 (13.9)	68 (10.5)	27 (69.2)

Abbreviations: HBV, hepatitis B virus; HCV, hepatitis C virus.

### Prevention of vertical transmission

Tenofovir, recommended for administration to pregnant women with high HBV‐DNA levels after 28 weeks of gestation to prevent the intrauterine transmission of HBV, was used in 42.1% (32/76) of HBe antigen–positive carriers (data not shown). Although the overall rate of elective cesarean delivery to prevent vertical transmission in HCV carriers was 1.5% (10/684), all 10 patients were carriers of high HCV‐RNA levels, and the cesarean delivery rate in carriers with high HCV‐RNA levels was 47.6% (10/21) (data not shown).

## DISCUSSION

This survey clarified several key points regarding pregnant hepatitis virus carriers in Japan. (1) The prevalence of pregnant hepatitis virus carriers was 0.2% for HBV (0.1% in Japanese women and 3.0% in non‐Japanese women) and 0.2% for HCV (0.2% in Japanese women and 0.3% in non‐Japanese women). (2) Only 60%–70% of pregnant carriers consulted internal medicine (i.e., hepatology specialists). A major concern is that the internal medicine consultation rate was low in teenagers diagnosed as HBV carriers before pregnancy (9.1%) and in women diagnosed as HCV carriers before pregnancy (10.5%). (3) In terms of preventing mother‐to‐child transmission, the use of tenofovir for HBV has become more widespread, and nearly 50% of pregnant HCV carriers with high HCV‐RNA levels underwent cesarean delivery for this purpose.

The overall prevalence of HBV carriers of 0.2% (0.1% in Japanese women and 3.0% in non‐Japanese women) is consistent with previous findings.^2^ Although inadequate measures for the prevention of vertical transmission and low vaccination coverage may have contributed, the present survey did not ascertain the specific nationalities of non‐Japanese individuals, making it difficult to conduct a detailed analysis of the reasons underlying the higher HBV carrier rate among non‐Japanese. Our survey also revealed a higher HBV carrier rate in Japanese teenagers. Although the present study was not designed to elucidate transmission routes in young women, sexual activity, cosmetic procedures involving skin penetration (e.g., tattoos and piercings), and medical interventions may contribute to the higher risk of HBV infection in this age group. Future studies need to examine these risk factors and reinforce effective preventive measures for young individuals. In addition, some infants targeted by vertical transmission prevention measures may have missed vaccination or not received appropriate follow‐up. Since 2016, infants born to non‐HBV carriers in Japan have also been administered the hepatitis B vaccine, and a decline in the prevalence of HBV carriers in younger generations is anticipated. The establishment of a system that verifies the proper implementation of HBV vertical transmission prevention measures, particularly for infants born to HBV carriers, is essential. The present results indicate that only 60%–70% of HBV carriers received internal medicine consultations. In particular, the internal medicine consultation rate for teenage HBV carriers diagnosed before pregnancy was extremely low at 9.1%. In response to the low consultation rates reported in the nationwide survey conducted by Tanaka et al. in 2016–2017,^3^ the government‐initiated subsidies for the cost of the initial detailed examination for women who test positive for the hepatitis virus during prenatal check‐ups. However, the present results obtained herein indicate that these measures have not led to an improvement in consultation rates. This may reflect inadequate knowledge of HBV among younger individuals as well as socio‐economic factors affecting healthcare utilization.^10^ Our survey also showed that only 64.6% of obstetric clinics had a policy of referring pregnant HBV carriers to internal medicine, highlighting the need for targeted educational activities. In addition, it may be necessary to conduct a survey of general internists, not just hepatologists, to raise awareness of the importance of follow‐up care for hepatitis carriers and, if necessary, educate them on this issue. Regarding the prevention of vertical transmission, the present results indicate that the maternal administration of tenofovir for HBV is becoming more widespread; 42.1% of pregnant HBe antigen‐positive carriers received tenofovir. However, the use of HBe antigen positivity alone is insufficient for risk stratification. Although our survey did not capture the rate of HBV‐DNA quantification testing, future practice needs to incorporate comprehensive risk assessments based on HBV‐DNA quantification in accordance with international standards, followed by appropriate antiviral therapy.

The overall prevalence of HCV carriers was 0.2%: 0.2% in Japanese women and 0.3% in non‐Japanese women, which is consistent with previous epidemiological findings. Among pregnant Japanese women aged ≤19 years, three were HCV carriers, two of whom had high HCV‐RNA levels. High viral loads in young women may be attributed to transmission via sexual contact, medical procedures, or cosmetic practices, similar to HBV. In addition, there may also be a possibility of missing vertical transmission or insufficient follow‐up. The internal medicine consultation rate for women known to be HCV carriers before pregnancy was only 10.5%, possibly because HCV infection is often asymptomatic in the acute phase and the need for management during pregnancy is emphasized less than that for HBV. In addition, low general awareness of HCV and the insufficient penetration of standardized guidelines among healthcare providers may have contributed to these findings. Regarding the prevention of vertical transmission, approximately 50% of pregnant HCV carriers with high HCV‐RNA levels underwent elective cesarean delivery to prevent vertical transmission. Although high HCV‐RNA levels may increase the risk of vertical transmission, the efficacy of selective cesarean delivery for this purpose remains controversial.^8^, ^9^ In the first place, there are no established criteria for high HCV‐RNA levels. In this survey, the overall rate of selective cesarean delivery among pregnant HCV carriers was 1.5%, but it increased to 47.6% in patients with high HCV‐RNA levels (≥6.0log IU/mL), suggesting that viral load‐based risk assessment and patient counseling may have influenced the decision regarding mode of delivery. Currently, antiviral therapy for HCV during pregnancy is not approved. However, even if vertical transmission of HCV occurs, there is a possibility that the virus may be naturally cleared, and it is possible to achieve cure efficiently with DAA. Therefore, cesarean delivery for the prevention of HCV vertical transmission based on viral load should be considered after providing patients with sufficient information. Further research is needed to confirm whether the rate of cesarean delivery for the purpose of preventing vertical transmission of HCV will decrease in the future.

The strengths of the present study include the large number of cases. Information was obtained from 1077 delivery facilities, and the number of deliveries at facilities that responded to this survey was 295,374, accounting for approximately 40% of the total number of deliveries in Japan in 2023, which was 727,277, encompassing 602 HBV carriers and 684 HCV carriers. However, several limitations need to be addressed. The response rate of 55.8% may have introduced a response bias. Moreover, since this was a cross‐sectional survey based on data from 2023, the long‐term impact of the HBV universal vaccination program implemented since 2016 has not been fully captured. In addition, we were unable to thoroughly evaluate or analyze the reasons behind the low internal medicine consultation and referral rates among pregnant hepatitis virus carriers, including factors related to individual facilities. The present survey did not capture details regarding the clinical management of internal medicine consultations for pregnant carriers or the extent of their subsequent follow‐up care.

In conclusion, the present study found that the prevalence of pregnant hepatitis virus carriers in Japan was approximately 0.2% for both HBV and HCV. Challenges remain, particularly the high HBV carrier rate and HCV carriers with high HCV‐RNA levels in teenagers. While vaccination and DAA therapies offer promise, only 60%–70% of pregnant hepatitis virus carriers receive appropriate internal medicine consultations, which indicate that the government's initiatives to increase follow‐up rates have not been fully effective.

## AUTHOR CONTRIBUTIONS

**Manabu Ogoyama:** Conceptualization; data curation; formal analysis; investigation; writing – original draft. **Shunji Suzuki:** Conceptualization; project administration; writing – review and editing. **Shin‐ichi Hoshi:** Data curation; formal analysis. **Akihiko Sekizawa:** Project administration; writing – review and editing. **Yoko Sagara:** Project administration; writing – review and editing. **Isamu Ishiwata:** Supervision. **Tadaichi Kitamura:** Supervision.

## CONFLICT OF INTEREST STATEMENT

All authors declare no conflicts of interest.

## Data Availability

Data sharing is not applicable to this article as no new data were created or analyzed in this study.
